# Acupuncture therapy and cognitive dysfunction in patients with type 2 diabetes: A protocol for systematic review

**DOI:** 10.1097/MD.0000000000026115

**Published:** 2021-05-28

**Authors:** Ting Pan, Xue Zhou, Xuefeng Li, Heran Wang, Meng Meng, Yiran Han, Xin Qian, Chunhai Chen, Xinhua Chen

**Affiliations:** a Department of Acupuncture and Tuina, Changchun University of Chinese Medicine; b Department of Acupuncture and Tuina, The Affiliated Hospital of Changchun University of Chinese Medicine, Changchun, China.

**Keywords:** acupuncture, cognitive impairment, protocol, systematic review, type 2 diabetes

## Abstract

**Background:**

With the aging of society, the incidence of type 2 diabetes (T2DM) is increasing every year, and there is a clear correlation between T2DM and cognitive dysfunction. Acupuncture therapy has been widely used in the treatment of T2DM, but there is no systematic review on the treatment of T2DM associated with cognitive impairment. Therefore, this study aimed to conduct a meta-analysis of acupuncture in the treatment of T2DM with cognitive impairment to clarify its efficacy.

**Methods:**

A structured and systematic literature search will be conducted in the following databases up to April 26, 2021: PubMed, Embase, Cochrane Central Register of Controlled Trials (CENTRAL), Web of Science (WOS), China National Knowledge Infrastructure (CNKI), Chinese Biomedical Literature Database (CBM), Chinese Scientific and Journal Database (VIP), and Wan Fang database (Wanfang). We will use the Review Manager 5.4 software provided by the Cochrane Collaborative Network for statistical analysis. We then assessed the quality and risk of the included studies and observed the outcome measures.

**Results:**

This meta-analysis further determined the beneficial effects of acupuncture on T2DM with cognitive impairment.

**Conclusion:**

The purpose of this meta-analysis was to explore the effect of acupuncture on patients T2DM with and cognitive impairment patients, and provide more options for clinicians and patients to treat T2DM with cognitive impairment.

**Ethics and dissemination:**

This systematics review will evaluate the efficacy and safety of acupuncture in the treatment of T2DM with cognitive impairment. Since all the data included were published, the systematic review did not require ethical approval.

**Registration number:**

CRD42021245681.

## 1 Introduction

Type 2 diabetes mellitus (T2DM), a metabolic disease characterized by a persistently high level of blood glucose, is caused by a progressive increase in insulin resistance and reduction in insulin secretion.^[1]^ Type 2 diabetes itself is a complex, age-related chronic disease, and its increasing prevalence is also of great public concern. Currently, 366 million people worldwide have diabetes mellitus, and this number is expected to reach 552 million by 2030.^[2]^ Compared with normal people without diabetes, diabetes was associated with an increased risk of cognitive impairment by 1.25 to 1.91 times.^[3]^ T2DM is a risk factor for cognitive dysfunction, including cognitive decline, mild cognitive impairment, and dementia. It is estimated that 20% to 70% of T2DM patients have cognitive impairment, and 60% of patients have a higher risk of dementia.^[4–7]^ Cognitive dysfunction can also worsen the compliance of diabetic patients worse, thereby aggravating diabetes, and the 2 form a vicious circle.

At present, the pathogenesis of T2DM with cognitive impairment is not fully understood, and we can actively seek for the prevention and treatment of T2DM with cognitive impairment from the aspects of glucose metabolism disorder, insulin resistance, abnormal adipocyte secreting factor, disruption of neuronal calcium homeostasis, and poor lifestyle.^[8–11]^ However, the mechanism of diabetic cognitive impairment is complex, and there are many related factors. The academic community has not reached a consensus in this regard, and further research and exploration are still needed. There is still a lack of targeted methods for the clinical treatment of cognitive dysfunction in patients with type 2 diabetes.^[12]^ Western medicine mainly focuses on lowering blood sugar, supplemented with drugs to improve cognitive impairment, such as pioglitazone, metformin, and irisin, to alleviate diabetes-induced central nervous system damage.^[13,14]^ As an important part of traditional medicine, acupuncture has a good therapeutic effect on diabetes and its complications, such as regulating blood sugar and delaying central nervous system damage.^[15,16]^ However, there is no systematic review of the efficacy of acupuncture in the treatment of diabetes mellitus with cognitive impairment. To the best of our knowledge, this is the first meta-analysis of randomized controlled trials (RCTs) to explore the effect of acupuncture on type 2 diabetes with cognitive impairment.

## 2 Methods

This protocol which has been reported is based on the Preferred Reporting Items for Systematic Reviews and Meta-Analyses Protocols (PRISMA-P) guidelines^[17]^ and the corresponding checklist used. This systematic review protocol was registered in the PROSPERO International Registry of Systematic Reviews (ID: CRD42021245681).

### 2.1 Inclusion criteria

#### 2.1.1 Types of studies

Only RCTs of acupuncture treating type 2 diabetes with cognitive impairment will be included in this study, without publication or language restrictions. Non-RCTs will be ruled out, including studies such as case reports, and studies without sufficient information about the randomized method or process.

#### 2.1.2 Types of interventions

Participants suffering from type 2 diabetes with cognitive impairment will be included, regardless of age, sex, nationality, and educational background.

#### 2.1.3 Types of interventions

##### 2.1.3.1 Experimental interventions

We will include acupuncture and acupuncture-related therapies (regular acupuncture, scalp acupuncture, auricular acupuncture, electroacupuncture, fire needling, intradermal needling, and catgut embedding acupuncture) to comprehensively describe the effects of acupuncture on patients with type 2 diabetes and cognitive impairment. Clinical trials with other forms of stimulation including moxibustion, bleeding therapy, cupping, laser acupuncture, pharmacoacupuncture, acupotomy, point injection, or acupressure, will be excluded. Additionally, limitations to intervention intensity, frequency, and duration were not involved.

##### 2.1.3.2 Control interventions

Studies that are interfered with no treatment, an acupuncture-like intervention will be considered as a control group. Additionally, studies comparing acupuncture plus the concomitant treatment with that treatment will also meet the control group including criteria. However, studies comparing acupuncture with other traditional chinese medicine treatments have not been conducted.

#### 2.1.4 Types of outcome measures

The primary outcome measures were fasting blood glucose, 2 hours postprandial blood glucose (2hG), and glycosylated hemoglobin. In addition, improvements in memory and learning ability, spatial disorientation, motor function, and better emotional management of depression or anxiety will be assessed. Relative validated scales such as Minimum Mental State Examination (MMSE), activity of daily living (ADL), and neuropsychiatric inventory (NPI), will be employed to evaluate physiological and psychiatric changes, and the second observation index mainly includes triglyceride (TG), cholesterol (TC), insulin secretion (INS), and so on.

#### 2.1.5 Types of studies

Randomized controlled clinical trials and quasi-randomized controlled trials will be included.

### 2.2 Search methods

We searched 8 electronic databases of PubMed, EMBASE, Cochrane Central Register of Controlled Trials (CENTRAL), Web of Science (WOS), China National Knowledge Infrastructure (CNKI), Chinese Biomedical Literature Database (CBM), Chinese Scientific and Journal Database (VIP), and Wan Fang database (Wanfang) to identify literature on RCTs for type 2 diabetes with cognitive impairment. We also searched the Chinese Clinical Trial Registry (ChiCTR) and ClinicalTrials.gov (www.ClinicalTrials.gov/) for in-progress trials with unpublished data. Table 1 presents the PubMed search strategy.

**Table 1:** Search strategy for the PubMed database.

Number	Terms
#1	Type 2 diabetes mellitus (all field)
#2	type 2 diabetes (all field)
#3	T2DM (all field)
#4	#1 OR #2–3
#5	Cognitive dysfunction (all field)
#6	Cognitive impairment (all field)
#7	Neurodegeneration (all field)
#8	#5 OR #6–7
#9	Acupuncture (all field)
#10	Needling (all field)
#11	acupoint (all field)
#12	Acupuncture treatment (all field)
#13	Acupunctue needling (all field)
#14	Scalp acupuncture (all field)
#15	Fire needling (all field)
#16	Ear acupuncture (all field)
#17	Intradermal needling (all field)
#18	Auricular acupuncture (all field)
#19	Electroacupuncture (all field)
#20	Catgut embedding (all field)
#21	Catgut embedding (all field)
#22	#9 OR #10–21
#23	Randomized controlled trial (all field)
#24	Controlled clinical trial (all field)
#25	Randomly (all field)
#26	Randomized (all field)
#27	Random allocation (all field)
#28	Placebo (all field)
#29	Double-blind method (all field)
#30	single-blind method (all field)
#31	Trials (all field)
#32	#23 OR #24–32
#33	#4 And #8 And #22 And #32

### 2.3 Data collections and analysis

#### 2.3.1 Selection of studies

**Figure 1:**
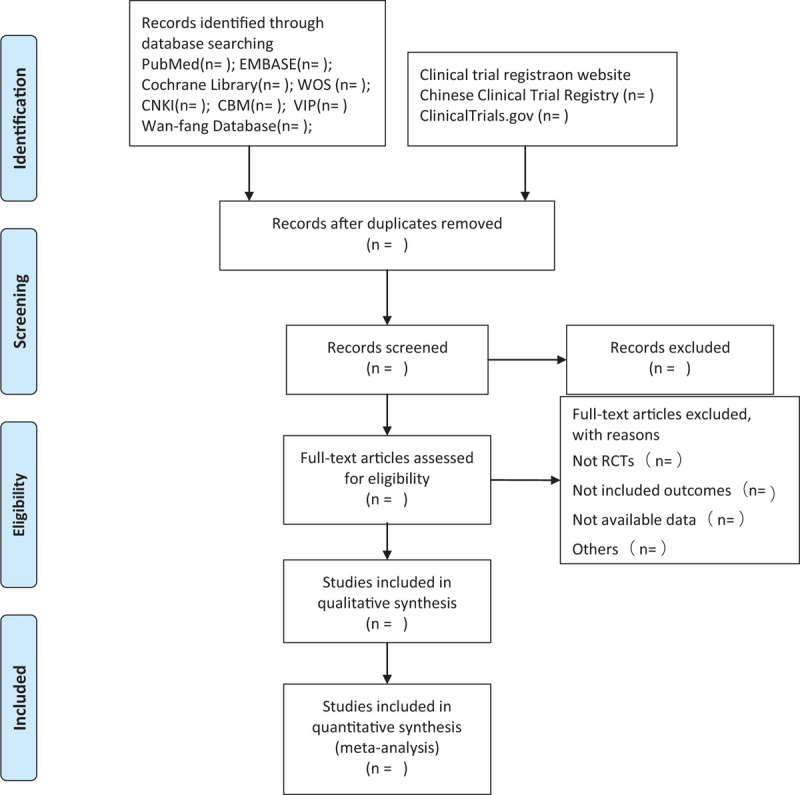
Flow diagram of study selection process.

To ensure that all reviewers have a comprehensive understanding of the purpose and process of this study, we will organize a group meeting to deliver relative information before conducting this study. Two authors (XL and XZ) will screen the titles and abstracts, respectively, to extract potentially eligible articles, and duplicated results will be excluded. Further identification will be carried out by reviewing the full text and analysis considerations to select eligible studies. Then, all reviewers will have a group discussion on the consistency of all the studies included, excluding and eliminating those that were not up to the theme topic until the final team consensus arrived. The selection process is fully elucidated in the following PRISMA flow diagram (Fig. 1).

#### 2.3.2 Data extraction and management

Then, 2 reviewers (RH and MM) will extract the title, first author, publication year, country, language, journal source; information of participants: sex, age, study design, sample size, intervention, type of measures, risk of bias assessment, and findings from included studies with Excel file. The results will be cross-checked by the 2 reviewers, and any disagreements will be resolved by consensus, with any ongoing differences in opinion being arbitrated by a third reviewer (HW).

### 2.4 Statistical analysis

We will use the Review Manager 5.4 software provided by the Cochrane Collaborative Network for statistical analysis. For continuous variables, the mean and SD of each study were obtained and pooled as mean difference (MD) or standardized mean differences (SMD) with a 95% confidence interval (CI). Statistical heterogeneity analysis was performed for the included clinical RCTs. The Cochrane *I*^2^ test was used for statistical analysis. When *I*^2^ was <50% or *P* > .05, it indicated that there was no statistical heterogeneity between the studies, and the fixed-effect model was selected to combine the effect amount; otherwise, the random effect model was adopted.

### 2.5 Methodological quality of assessment

The literature quality of this study was evaluated using the bias risk table proposed by the Cochrane Collaborative Network. The risk table includes 6 items: random sequence generation mode, whether to use allocation concealment, whether to blind the subjects and intervention providers, whether to blind the results evaluators, whether the results data are complete, whether to select the results report, and other bias sources. The criteria used to assess the risk of bias were “low risk,” “high risk,” and “unclear.” In this process, 2 evaluators independently evaluated methodological quality. In cases of disagreement, the third author intervened.

### 2.6 Assessment of heterogeneity

Before the combination of effect size, we will use Stata to assess the available study and patient characteristics to ensure similarity and to investigate the potential effect of heterogeneity on effect estimates. When interstudy heterogeneity exists, a random effects model is used. For comparison of each pair, heterogeneity was assessed by the statistic *I*^2^. When *I*^2^ > 50%, this indicates that there is heterogeneity between studies, and the source of heterogeneity should be further investigated. When *I*^2^ < 50%, interstudy heterogeneity was considered to be small, or there was no obvious heterogeneity.

### 2.7 Assessment of publication bias

If there are >10 trials in accordance with the study, we can use Rev Manager 5.4 software to draw and analyze the funnel chart, and use the funnel chart to evaluate the potential publication bias.

### 2.8 Grading the quality of evidence

Grading of recommendations assessment, development, and evaluation reliability study (GRADE) will be implemented to assess the quality of evidence. Based on the risk of bias, inconsistency, imprecision, indirection, and publication bias, GRADE grades evidence quality into 4 levels: high, medium, low, and very low.

### 2.9 Ethics and dissemination

Formal ethical approval was not required for this protocol. Because nothing of the information will be obtained from an individual participant, the systematic review does not require ethical approval.

## 3 Discussion

Type 2 diabetes is now considered an independent risk factor for mild cognitive impairment.^[18]^ Many studies have shown that poor glycemic control is strongly associated with the development of cognitive dysfunction.^[19,20]^ Acupuncture originated in ancient China and has been widely applied in the clinic for a long time. It is an important component of Eastern medicine. With the further study of TCM on type 2 diabetes and cognitive impairment, acupuncture can effectively improve the blood glucose level of patients and enhance the treatment effect. In recent years, many basic studies have also shown that acupuncture may improve the cognitive function of patients by improving the abnormal state of the cholinesterase system, regulating the synaptic plasticity of the hippocampus, and alleviating the inflammatory response of the central system.^[21,22,23]^ This systematic review will evaluate the efficacy and safety of acupuncture in the treatment of T2DM with cognitive impairment. The significance of a review and meta-analysis is to evaluate its effect in a larger sample, and to summarize the current research results to further provide advice on clinical research, which will have a positive significance in protecting the cognitive impairment of diabetic patients.

## Author contributions

**Conceptualization:** Ting Pan, Chunhai Chen.

**Data curation:** Xue Zhou, Xuefeng LI.

**Formal analysis:** Heran Wang, Meng Meng, Yiran Han.

**Funding acquisition:** Xinhua Chen.

**Investigation:** Xue Zhou, Xuefeng Li.

**Methodology:** Ting Pan, Chunhai Chen.

**Supervision:** Xinhua Chen.

**Validation:** Heran Wang, Yiran Han, Xin Qian.

**Writing – original draft:** Ting Pan.

**Writing – review & editing:** Ting Pan, Xinhua Chen, Chunhai Chen.
